# Editorial: Heterogeneity of ILC2s

**DOI:** 10.3389/fimmu.2023.1230569

**Published:** 2023-06-26

**Authors:** Hiroki Kabata, Koichi Fukunaga

**Affiliations:** Division of Pulmonary Medicine, Department of Medicine, Keio University School of Medicine, Tokyo, Japan

**Keywords:** group 2 innate lymphoid cells, diversity, IL-33, tissue-specific, allergy

In 2010, novel subsets of lymphocytes were discovered in various tissues and were respectively named natural helper cells (1), nuocytes (2), and innate helper type 2 cells (3). Due to the similarities in transcription factors and cytokine production, these cells were subsequently consolidated into a unified group termed group 2 innate lymphoid cells (ILC2s) in 2013 (4). Further research has uncovered that ILC2s exhibit diverse functions and participate in allergic inflammation, parasite infections, tissue repair, and metabolic homeostasis (5). However, as our understanding of ILC2s deepens, their heterogeneity is once again attracting attention. For example, in 2015, a distinct subset of ILC2s known as inflammatory ILC2s was identified (6). In 2016, the first single-cell RNA-seq analysis of ILC2s demonstrated that intestinal ILC2s can be further categorized into multiple subgroups (7). During this period, it was also discovered that ILC2s possess the ability to modify their phenotype in response to the surrounding cytokine environment, which renders it uncertain whether there exist discrete subsets of ILC2s or a mixture of ILC2s at various stages of activation or development (8). Nevertheless, numerous recent studies have furnished evidence for the varied expression of tissue-specific markers, cytokine secretion profiles, and transcription factors in ILC2s (9). These findings imply that ILC2s exhibit distinct characteristics in different tissues and pathologies, potentially playing intricate roles *in vivo*.

To shed light on the complex heterogeneity of ILC2s, this Research Topic presents a compilation of original research articles and review articles under the title “Heterogeneity of ILC2s.” Firstly, Kogame et al. discuss the latest advancements in understanding the differentiation and maturation process of ILC2s, which are closely associated with their heterogeneity. Secondly, ILC2s are tissue-resident cells with distinct characteristics in each tissue. Misawa et al. summarize the diversity of ILC2s in adipose tissue, Kobayashi et al. in skin, Sunaga et al. in the intestinal tract, and Asaoka et al. in the lung. These review articles provide readers with up-to-date knowledge on the diversity of ILC2s in each specific tissue. Furthermore, ILC2s exhibit functional heterogeneity. Ikutani and Nakae and Nakagome and Nagata summarize the roles of ILC2s in cancer, obesity, cardiovascular disease, and viral infections, respectively. Matsuyama et al. outline therapeutic strategies targeting ILC2s in asthma. These review articles highlight the functional diversity of ILC2s in the context of various disease processes. Lastly, Topczewska et al. using *Nmur1* ^iCre-eGFP^ mice generate ILC2-specific conditional knockout mice. Their work elegantly uncovers the role of IL-33 in ILC2s across multiple tissues.

This Research Topic not only presents the latest discoveries in basic research but also highlights the potential for future treatment strategies. Targeting the activation or suppression of specific subgroups of ILC2s holds promise as a novel approach in treating allergic and immune-related diseases. Advancing our understanding of the molecular mechanisms underlying ILC2 heterogeneity will be crucial for developing more precise control methods and identifying therapeutic targets, ultimately paving the way for personalized therapeutic strategies in the future.

## Author contributions

All authors listed have made a substantial, direct, and intellectual contribution to the work and approved it for publication.
